# Exploring risk and protective urban environmental factors on mental health through exposure network mapping

**DOI:** 10.21203/rs.3.rs-7155816/v1

**Published:** 2025-08-06

**Authors:** Na Luo, Zhengyi Yang, Ming Song, Shiqi Di, Congying Chu, Weiyang Shi, Yuyanan Zhang, Weihua Yue, Jing Sui, Vince Calhoun, Tianzi Jiang

**Affiliations:** Institute of automation, Chinese academy of sciences; Institute of automation, Chinese academy of sciences; Institute of automation, Chinese academy of sciences; University of Chinese Academy of Sciences; Institute of automation, Chinese academy of sciences; Institute of automation, Chinese academy of sciences; Peking University Sixth Hospital, Peking University Institute of Mental Health; Peking University Sixth Hospital, Peking University Institute of Mental Health; Beijing Normal University; Georgia State University, Georgia Institute of Technology, Emory University; Institute of automation, Chinese academy of sciences

**Keywords:** urbanicity, exposome, human brain networks, stress, sleep, psychiatry

## Abstract

**BACKGROUND:**

Urbanicity has been revealed to carry a higher risk of experiencing mental health issues. However, the factors in urban environments that pose risks or protective impacts on mental health remain unclear.

**METHODS:**

Based on eight literature-based datasets and one validation dataset, this study introduced a new technique termed exposure network mapping (ENM) to explore the impacts of urbanicity on brain networks, identify the potential risk urban environmental factors, and examine whether healthy lifestyle habits may provide protective effects on mental health.

**RESULTS:**

Using ENM, this study consolidated existing heterogenous coordinates of urbanicity into a common, significant and replicable network, which primarily located in middle frontal gyrus, orbital gyrus and anterior cingulate gyrus. When conducting ENM analysis using coordinates of five representative factors (i.e., air pollution, noise pollution, income, stress, and green space), only seeds derived from stress significantly converged to a common network, highlighting orbital gyrus, caudate, anterior and middle cingulate gyrus, hippocampus and middle frontal gyrus. The ENM-stress map further exhibited the highest correlation with both the ENM-urbanicity map(*r* = 0.77) and a transdiagnostic map(*r* = 0.72). Finally, ENM analysis using coordinates of sleep also enriched in a distinct common network, featuring middle cingulate gyrus, orbital gyrus, caudate and putamen, which concurrently demonstrated strong correlations with urbanicity(*r* = 0.75), stress(*r* = 0.80), and the transdiagnostic map(*r* = 0.55).

**CONCLUSION:**

This study highlights the potential risks of urbanicity and stress on brain networks, as well as the protective role of healthy habitats—particularly sleep—in safeguarding mental health, which may offer new insights for preventing mental health issues in urban environments.

## Introduction

According to the latest report of World Urbanization Prospects, more than half of the world’s population, equivalent to 3.9 billion people, currently reside in urban areas[1]. Although city dwellers, on average, are wealthier and receive improved sanitation, nutrition and health care, rapid urbanization can lead to environmental issues and social problems. Existing studies have revealed that these urban environmental exposures may carry a higher risk of experiencing mental health issues[2, 3], therefore it is important to explore what impacts urbanicity may leave on our brain networks.

Meanwhile, urbanicity is not only a demographic factor but also includes four dimensions (ecosystems, lifestyle, social and physical-chemical factors) that constitute the whole picture[4, 5]. For example, urbanicity can increase urban land use and anthropogenic emissions, which in turn can impact the concentrations of air pollutants as well as the associated health risks[6]. The rapid pace brought by urbanicity keeps people in a constant state of stress, exerting a significant impact on their physical and mental well-being[7]. However, the differential impact of various exposure factors has yet to be investigated. Exploring which exposure factor shows the most significant influence on the human brain may contribute to urban planning and policy making.

Given the significant risks associated with urban living, are there any viable approaches to mitigate the effect of urbanicity on the human brain? Maintaining certain habits, like daily coffee intake, regular physical activity, or a good sleep habitat over a long term, have been revealed to affect our brain[8]. Among all the habitats, sleep is likely to support a fundamental need of the organism. It plays an important role in memory processing, brain plasticity, and regulating emotional brain reactivity[9, 10]. A meta-analysis of randomized controlled trials reported that the effects of an intervention on sleep helped improve composite mental health and seven specific mental health difficulties[11]. However, whether healthy sleep habits may act as an available manner to mitigate the effects of urbanicity on human brain remains unexplored.

Considering the challenges of collecting data across different sites and the diversity of exposure factors, existing neuroimaging studies on urbanicity either have a relatively small sample size or only assess a single exposure factor. Results from these studies are also controversial, exhibiting high heterogeneity. To overcome these difficulties, this study proposed a new technique termed ‘exposure network mapping’ (ENM) inspired by lesion network mapping (LNM) method[12–14]. It helps largely overcome the heterogeneity and sparsity in findings and improves determination of convergence in common neuroimaging networks compared to traditional activation likelihood estimation (ALE) method[13, 15]. LNM has been extended by replacing brain lesions with coordinates of brain structural atrophy[16], brain stimulation sites[17] and task-derived activation[18] as seeds. However, slight variations in brain networks of healthy participants caused by environmental factors have not been explored via this technique. Our proposed ENM technique was designed to use the reported coordinates of exposure factors (i.e., urbanicity, air pollution, *etc*.) as seeds to identify the brain networks of exposure effects in the normative connectome.

In this study, to systematically answer the above questions, 1) we conducted ENM analysis to study the impact of urbanicity on brain networks and replicated the main results on an independent magnetic resonance imaging (MRI) dataset, as well as a transdiagnostic map for six psychiatric illnesses. 2) we then computed ENM analysis on five sub-exposome factors, including air pollution, noise pollution, greenspace, stress, and household income to explore which factor may present the highest spatial association with urbanicity and the transdiagnostic map. 3) we finally computed ENM results using sleep coordinates to investigate the relationship between impacts of good lifestyle habits and the risk urban environmental factors on mental health.

## Methods

### Urbanicity study selection

We searched the PubMed databases to include urbanicity-related neuroimaging studies in the latest ten years (from November 2014 to November 2024) with search terms consisted of ‘((urbanicity OR urban OR urbanization) AND (magnetic resonance Imaging OR neuroimaging OR grey matter OR voxel OR cortical) AND (brain) AND (human))’. The main inclusion criteria were as follows: (1) reported Talariach or Montreal Neurological Institute (MNI) coordinates on grey matter, (2) only involved healthy participants, (3) made use of MRI techniques, (4) written via English language, (5) human studies. A total of 23 urbanicity studies including 6,274 subjects were included in our study (Dataset 1 in Table 1, Table S2). The coordinates reported in Talairach space were non-linearly transformed into MNI space [19].

### ENM analysis using coordinates of urbanicity

**Figure 1:**
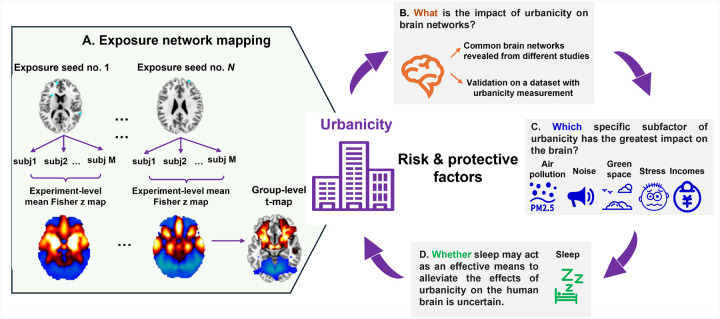
The whole analysis framework in this study. A. We first proposed an exposure network mapping (ENM) method, which used the reported coordinates of existing studies as input seeds. B. Upon using ENM method, we explored the impact of urbanicity on brain networks and replicated the main results on another neuroimaging dataset, as well as a transdiagnostic map for six psychiatric illnesses. C. We then computed ENM analysis on five sub-exposome factors, including air pollution, noise pollution, greenspace, stress, and household income to explore which factor may present the highest spatial association with urbanicity and the transdiagnostic map. D. We finally computed ENM results using sleep coordinates to investigate the relationship between impacts of good lifestyle habit and the risk urban environmental factors on mental health.

To explore whether slight variations in brain networks of healthy participants caused by environmental factors can be also extracted by a strategy similar to LNM[12], we extended to propose a new technique termed ENM to determine the common network derived from different urbanicity and other exposure studies (Fig. 1). First, a 3-mm-radius sphere centred on each coordinate reported in each study was created. We then merged these spheres of the same study to obtain a combined seed. A normative connectome of healthy controls from the Genome Superstruct Project (GSP)[20, 21] was used to compute the resting-state functional connectivity between each study-level combined seed and the rest of the brain. Each of the resulting subject-level *r* maps was transformed to a Fisher *z* map via Fisher’s *z* transformation. We then averaged the subject-level Fisher *z* maps of each study to create an experimentlevel mean Fisher *z* map. These study-level mean Fisher *z* maps were compared against zero using a one-sample t-test to identify brain regions significantly connected to the selected environment factors[18].

### Validation of another independent dataset

To validate the brain networks achieved from ENM analysis, we first included 140 healthy participants (Table 2) collected from the Chinese local community[22]. Samples were divided into two urbanicity groups: born and raised in rural areas from birth to 18 years old (Group I); born and raised in cities (Group II). The recruitment details are listed in the supplementary files. Structural MRI (sMRI) for all participants were acquired by a 3.0T GE Discovery MR750 scanner in the Center for MRI Research, Peking University. The T1-weighted structural imaging were preprocessed with a unified model in SPM12 (www.fil.ion.ucl.ac.uk/spm/software/spm12), including image registration, bias correction, tissue classification, spatial normalization to the standard MNI space and smooth. After preprocessing, the three-dimensional T1 brain image (3mm×3mm×3mm) of each subject was reshaped into a onedimensional vector and stacked, forming a subject-by-voxel matrix (140×73965). The ENM-urbanicity map was projected onto the subject-by-voxel matrix, generating a set of subject-specific weights that corresponded to the extent that a given subject’s data could be represented by the ENM-derived spatial map, as in the literature [23]. This was accomplished by multiplying the subject-by-voxel matrix by the pseudoinverse of the ENM-urbanicity map. These generated weights were then evaluated for group difference and association with the urbanicity score to assess whether the ENM-urbanicity map could be generalized to another independent cohort.

### ENM analysis using coordinates of each subfactor

According to Vermeulen et al.[4], exposure factors primarily included four domains: ecosystems, social, physical-chemical and lifestyle. As using ENM methods requires a sufficient number of MRI studies reporting coordinates of the affected brain regions, we selected five representative exposure factors from the four domains, including air pollution, noise pollution, greenspace, household income, psychological and mental stress (Figure S1). After a thorough literature search of the latest ten years (Table S1), we applied the same pipeline used for urbanicity to compute ENM results for each factor. We then assessed the spatial correlations between the ENM results of urbanicity and those of each factor, as well as among sub-factors. Furthermore, we identified brain regions that were shared across strongly correlated factors.

### Correlation with transdiagnostic network

To better explore how urbanicity may contribute to a higher risk of psychiatric disorders, we computed the spatial correlation between the identified ENM-urbanicity map and a recently published transdiagnostic network for six psychiatric illnesses [14]. This transdiagnostic network is constructed using coordinate and lesion network mapping across schizophrenia, bipolar disorder, depression, addiction, obsessive-compulsive disorder and anxiety.

### ENM analysis using coordinates of sleep

In our review of key factors within the lifestyle dimension, including sleep, physical activity, and coffee consumption habits, we found that sleep was the most extensively studied factor in relation to human brain networks. To ensure sufficient coordinates for ENM analysis, we consequently selected sleep as our primary focus for investigation. The search terms are listed in Table S1. After computing the ENM network based on sleep coordinates of the latest ten years, we analyzed whether existing heterogenous results of sleep could be enriched into a distinct common network. Furthermore, we examined the association between the derived ENM-sleep network and the ENM-urbanicity network, as well as each ENM map of the five exposure factors and the transdiagnostic map, which could provide a view of whether favorable habitat factors (e.g., quality sleep) might provide protective benefits against mental health disorders.

## Results

### ENM analysis of urbanicity

**Figure 2:**
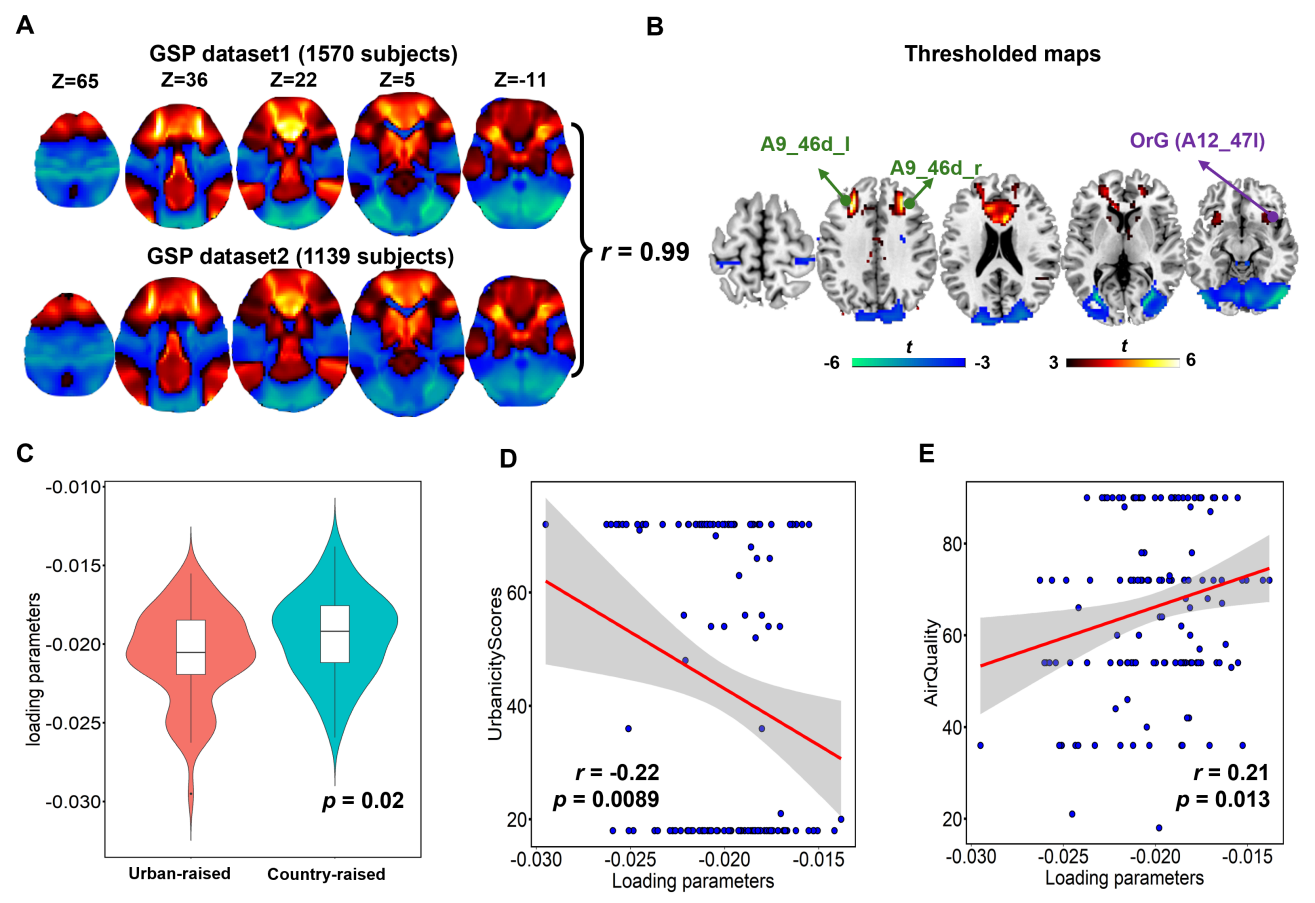
ENM analysis using urbanicity coordinates. A. Utilizing a large resting-state normative connectome (*n*=1570 subjects) and a validate normative connectome (*n*=1139 subjects) from GSP, ENM derives the underlying brain network of urbanicity effect on human brain. B. The peak cortical region (|*T*| > 3) highlighted the middle frontal gyrus (A9_46d_I, A9_46d_r) and orbital gyrus, insula, basal ganglia, and the visual network. C. After projecting the identified ENM-urbanicity map to another independent neuroimaging dataset, a significant group difference was observed between country-raised participants and city-raised participants. Further association analysis presented a significant positive correlation with the urbanicity score (D) and a significant negative correlation with air quality (E).

A total of 23 urbanicity studies including 6274 subjects were included in our study (Dataset 1 in Table 1, Table S2). Utilizing a large resting-state normative connectome (*n* = 1570 subjects) from GSP, we performed ENM analysis to determine whether highly inconsistent results across the 23 studies localized to a common network. The uncorrected resulting ENM-urbanicity maps are presented in Fig. 2A. Since the GSP dataset consists of two sub-datasets, which include similar individuals but collected on different days, we used the dataset with a larger number of individuals as the main dataset. When conducting ENM analysis on the smaller dataset (*n* = 1139), the resulting ENM-urbanicity map closely resembled the discovery dataset (Fig. 2A, r = 0.99). After thresholded the resulting map using |*T*| >3 (corresponding to a voxelwise FDR-corrected P < 0.05), middle frontal gyrus, orbital gyrus, anterior cingulate gyrus, insula, basal ganglia, and the visual network are still survived as shown in Fig. 2B. We further mapped the thresholded ENM-urbanicity results onto the Brainnetome Atlas[24] to identify the exact brain regions (Table S3).

### Validation of another independent dataset

When projecting the identified ENM-urbanicity map onto the preprocessed urbanicity dataset, a significant group difference (*p* = 0.02, Fig. 2C) was observed between participants from Group I (born and raised in rural areas from birth to 18 years old) and participants from Group II (born and raised in cities). Further association analysis presented a significant positive correlation (Fig. 2D, p = 0.0089) with the urbanicity score as defined in [22] and a significant negative correlation with air quality (Fig. 2E, p = 0.013).

### ENM on five exposure factors

The included studies for each factor are listed in Table 1 and Table S4-Table S8. Regarding the physical-chemical dimension, we selected air pollution and noise pollution as the representative factors. A total of 14 air pollution studies with 15 experiments, encompassing 5120 participants was included in this study (Table S4). Among these, one study conducted two separate analyses—one focusing on pregnancy exposure and the other on childhood exposure. For the noise pollution, we included 8 studies with 8 experiments including 515 participants (Table S5). Regarding the ecosystems dimension, a total of 6 green space studies with 6 experiments, encompassing 601 participants was selected as the representative factor (Table S6). Regarding the social dimension, we included the household incomes and psychological and mental stress as two representative factors. A total of 10 studies with 10 experiments including 1361 participants were included for household income (Table S7). For stress, we included 40 studies with 40 experiments including 5398 participants in the study (Table S8).

**Figure 3:**
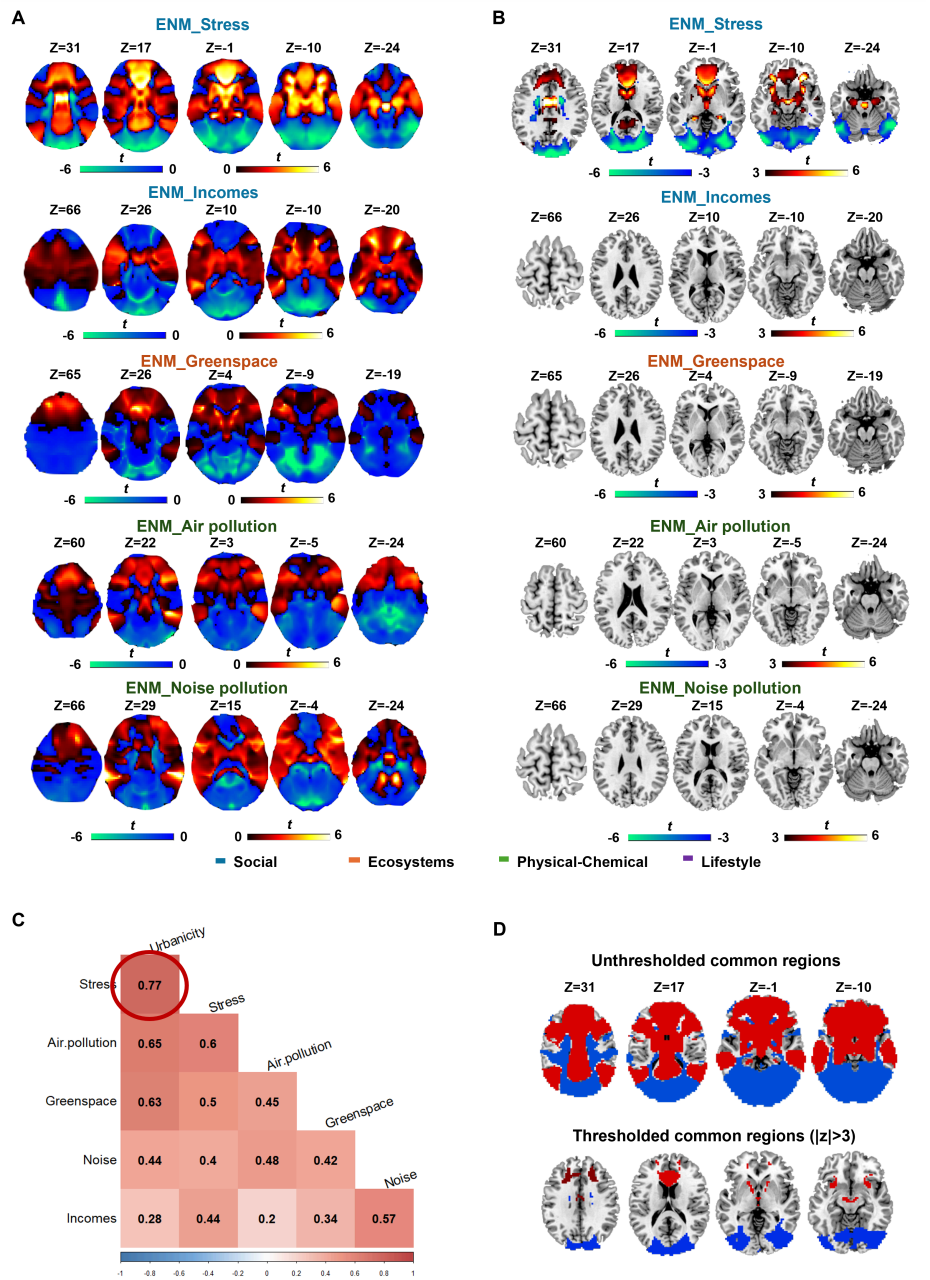
ENM on five exposure factors. A. The ENM results of five sub-exposure factors, including stress, incomes, green space, air pollution and noise pollution. B. The significant survived brain regions which passed the voxelwise FDR correction. C. The spatial correlation between each pair of different exposure factors. D. The overlapped regions between the ENM-stress map and the ENM-urbanicity map.

ENM analysis was then performed on each factor, and the corresponding results are presented in Fig. 3A. Among all factors, we found that only the previously reported heterogeneities associated with stress can be enriched to a significant common network using |*T*| >3 (corresponding to a voxelwise FDR-corrected P < 0.05). The key brain regions highlighted brain regions including the orbital gyrus, caudate, anterior and middle cingulate gyrus, hippocampus, and the middle frontal gyrus (Fig. 3B, Table S9). We then computed spatial correlations among the maps of different factors, which indicated that stress exhibited the strongest correlation with urbanicity (*r* = 0.77), followed by air pollution (*r* = 0.65) and green space (*r* = 0.63) as shown in Fig. 3C. The common region between stress and urbanicity primarily located in the middle frontal gyrus (A9_46d_I, A9_46d_r), the orbital gyrus, anterior cingulate gyrus and the visual network (Fig. 3D, |*T*| >3).

### The association with psychiatric illness

To examine the associations between these exposure factors and psychiatric disorders, we computed the correlations between the map of each factor and a transdiagnostic network for six psychiatric illnesses. The results revealed that stress exhibited the strongest correlation with the transdiagnostic commonality map (*r* = 0.72), followed by urbanicity (*r* = 0.58), air pollution (*r* = 0.53) and green space (*r* = 0.52).

### Sleep modulates the influences caused by urbanicity

**Figure 5:**
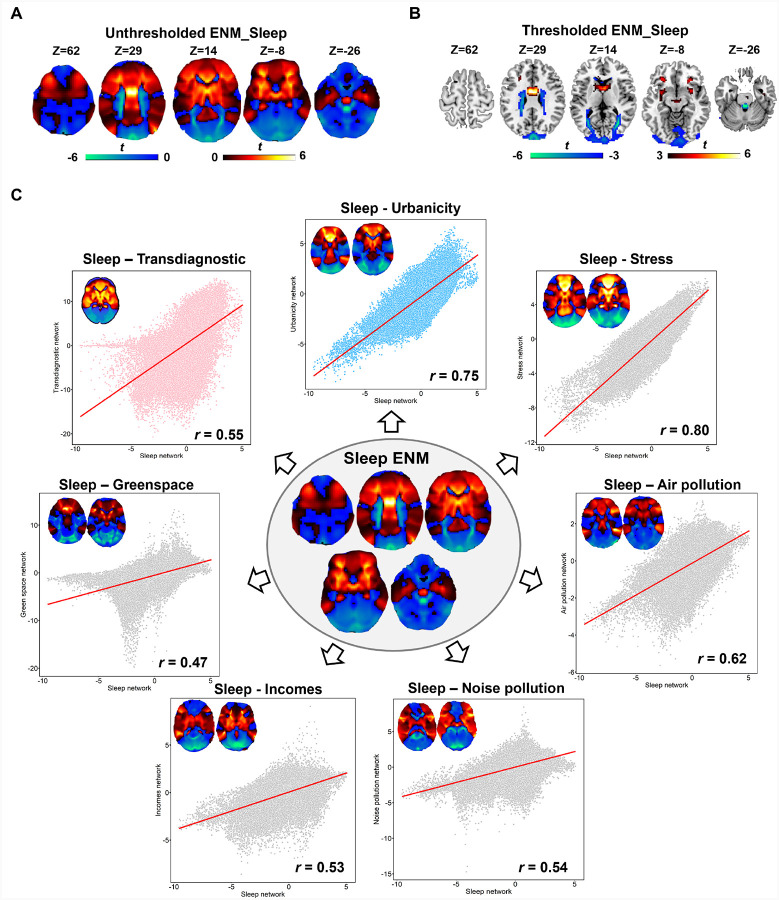
ENM analysis using sleep coordinates. A. The uncorrected ENM map of sleep. B. The significant regions passed voxelwise FDR correction. C. The association between the map of sleep and other factors. The inner figure indicates the ENM results on sleep. The outer figures represent the correlation between the ENM-sleep map and the ENM-urbanicity, the ENM maps of five-exposure factors, as well as the transdiagnostic maps.

After conducting a literature search, a total of 40 studies with 40 experiments including 5398 participants were included in our study (Table 1, Table S10). ENM analysis using these coordinates revealed the uncorrected ENM_sleep map as shown in Fig. 5A. After voxelwise FDR correction with |*T*| >3, the middle cingulate gyrus, the orbital gyrus, the caudate and putamen still survived (Fig. 5B, Table S11), indicating that previously reported neuroimaging heterogeneities in sleep research can be enriched to a common network. When assessing the relationship with other factors, sleep exhibits a remarkably strong correlation with urbanicity (r = 0.75), followed by stress (r = 0.80) and air pollution (r = 0.62). The association between sleep network and the transdiagnostic map was also significant (r = 0.55).

## Discussion

In this study, we introduced a new technique termed ENM to comprehensively explore the risk and protective urban environmental factors on mental health. The environmental-sensitive heteromodal association regions and the subcortical regions are significantly associated with urbanicity, which primarily encodes reward processing, emotional regulation and cognitive functions. Among the five subfactors of urbanicity, social factors (i.e., stress) are more indicative of the impact of urbanicity on the human brain compared to natural factors (i.e., air pollution). Intriguingly, maintaining healthy habits, like sleep, may help alleviate the negative effects of urbanicity and reduce the risks of experiencing mental health issues. These results provide important new information for understanding the effects of urbanicity on human brain networks and how to maintain brain health in the context of urbanization trends.

### What is the impact of urbanicity on brain networks?

After applying the proposed ENM method on urbanicity seeds, the peak resulting regions are primarily located in the middle frontal area (A9_46d_I, A9_46d_r), the orbitofrontal gyrus, insula, anterior cingulate gyrus, striatum, and the visual network. These regions are responsible for reward-related function and emotional function [25–27]. Our findings are consistent with a recent data-driven study which also reported that an environmental profile of social deprivation, air pollution, street network and urban landuse density was associated with brain regions responsible for reward processing[28]. Children who lived in more urban areas were revealed to be significantly more likely to exhibit behavioral and emotional problems in previous study[29]. Furthermore, most of these regions are primarily located in heteromodal association cortices, which demonstrate the most rapid expansion during human brain evolution[30], the greatest postnatal enlargement, and a low maturation rate[31, 32]. These indicate that these regions are exposed to environmental factors for a longer duration, resulting in a higher probability of being affected and more severe impacts.

In addition, impairments of these regions have been previously revealed to be associated with many mental disorders, including schizophrenia[33, 34], major depressive disorder[35, 36], and bipolar disorder[37, 38]. Consistently, we also observed a significant correlation between these regions and a transdiagnostic network for six psychiatric illnesses. The shared brain networks between urbanicity and mental disorders may explain why urbanicity is linked to a higher risk for mental illness. Moreover, after thresholding the ENM-urbanicity map, the middle frontal gyrus (A9_46d_I, A9_46d_r) and orbitofrontal gyrus (A11I_r, A12_47I_r) remain as the most significantly distinct regions on the cortex. Interestingly, these two regions are so far the two primary targets used for transcranial magnetic stimulation (TMS) treatment in depressive disorders[39–42].

### Which specific subfactor of urbanicity has the greatest impact on the brain?

Among the five exposure factors, stress has been ranked as the factor showing the highest association with urbanicity. Previous studies have found that increased social threat, a harsh and unpredictable environment, perceptions of neighborhood problems, social isolation, conditions of chaos, and commuting stress all contribute to increased urban stress[43, 44]. Several studies from both crosssectional and longitudinal studies suggest that stress exposure impacts reward-related regions and its function[45, 46], as highlighted by urbanicity. Moreover, stressrelated brain activation in regions important for emotion regulation were revealed to be associated positively with green space and associated negatively with air pollution and noise pollution[47]. There is a body of literature stating heavy stress may impact the ability to effectively regulate emotions[48]. In addition, stress also exhibited highest association with the transdiagnostic map for six psychiatric disorders. This is supported by existing studies, which indicated that chronic work stress may amplify the disability associated with psychiatric disorders and chronic physical conditions based on a large dataset analysis (*N* = 22,118) [49].

### Whether sleep may act as an effective means to alleviate the effects of urbanicity on the human brain is uncertain.

The final ENM analysis on sleep further highlights a strong association between sleep habits and urbanicity (*r* = 0.75), as well as stress (*r* = 0.80) and the transdiagnostic maps (*r* = 0.55). This result suggests that sleep may serve as a viable means of regulating the impact of urbanicity on the brain in its natural state. Existing study revealed that a 30-minute delay in school start time was associated with significant improvements in adolescent’s mood and health[50], predicting better academic performance[51]. Improved sleep quality is revealed to help alleviate stress in healthy subjects[52, 53]. Promotion of cognitive-behavioral therapy for insomnia patients improves sleep quality which produces lower general stress, lower depressive symptom severity, and better global health[54]. Furthermore, a study conducted on a significant sample of adults from the UK Biobank has revealed that adopting good sleep habits can have notable benefits in terms of slowing down cognitive decline, reducing the risk of mental illnesses and dementia[55]. Krause et al.’s recent review paper also points out sleep intervention is an underappreciated and novel target for disease treatment or prevention[56]. Certainly, the strong correlation between sleep and urbanicity also suggests that urbanicity or stress may impact sleep. However, the factors influencing sleep disorders are numerous, which is another topic.

### Considerations on the ENM Method: Unveiling Subtle Changes in Brain Networks

All the above results are achieved using our proposed ENM method. This is the first time used such a strategy to explore slight variations in brain networks of healthy participants caused by exposure factors instead of seeds from visible damage, abnormality or activation. The derived ENM-urbanicity map showed explainability in behavioral scales and has been replicated by another independent MRI dataset, which indicates the proposed method could capture the subtle changes in the brain. Further studies could also apply this method to measure the influences of more exposure factors on the human brain. In addition, this method can also be extended to diffusion tensor imaging data. Structural connectivity based on experimental-level seeds can be computed to identify covariation in structural networks across different studies.

### Limitations

Although our study revealed several interesting findings, it has the following limitations: 1) While we observed significant correlations among urbanicity, stress, sleep, and psychiatric disorders, this work remains correlational. Future experimental studies are needed to establish causal relationships between these factors. 2) Sleep was selected as the sole representative behavioral habit, though other habits (e.g., physical activity, social engagement, dietary patterns) may also link urbanicity to mental health. Future research may employ ENM analysis to investigate these relationships when sufficient studies reporting impaired coordinates for these factors are available. 3) We found no significant effects for air pollution, noise pollution, incomes, or green space. This may either be due to the high heterogeneity preventing convergence onto a common network, or the limited numbers of studies on these factors. Future studies could conduct further ENM analysis on these factors when more studies become available.

## Conclusion

This study introduces a new technique termed ENM to systematically investigate the influences of urban environmental factors on human brain networks. Among all the selected factors, only the existing heterogenous coordinates of urbanicity, stress, and sleep were successfully enriched to three common networks, highlighting regions such as the orbitofrontal cortex, anterior cingulate cortex, and striatum. Moreover, these three factors exhibited significant correlations with each other and were all associated with the transdiagnostic map for six psychiatric illnesses. These findings suggest that stress is a distinct feature of urbanicity, while maintaining good sleep habits may help manage stress and protect individuals from experiencing mental health issues in the context of rapid urbanization trends.

## Supplementary Material

This is a list of supplementary files associated with this preprint. Click to download.

- 2025ENMurbanicitysupplementary.docx

## Figures and Tables

**Figure 4:**
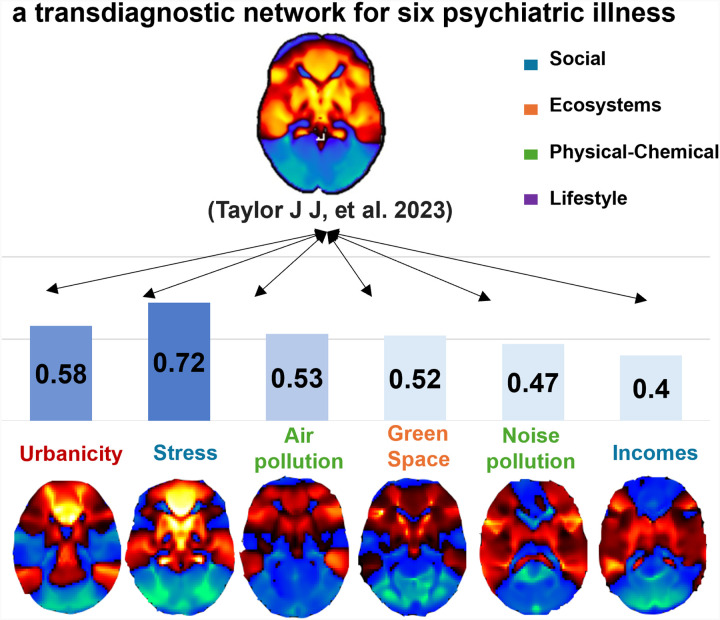
The correlation between maps of each exposure factor and a transdiagnostic network for six psychiatric illnesses.

**Table 1:** Descriptive statistics of the exposome factors used for ENM analysis

Exposure domain	Factors	Studies	Experiments	Participants	Foci
Urbanicity	Urbanicity	23	23	6274	149
Physical-chemical	Air pollution	14	15	5617	116
	Noise pollution	8	8	515	59
Ecosystems	Green space	6	6	601	30
Social	Household incomes	10	10	1361	70
	Psychological and mental stress	37	37	4066	236
Lifestyle	Sleep	40	40	5398	340

**Table 2:** Demographic information of Chinese and European validation dataset

Resource	Subjects	Age (year)	Gender(F/M)	Education
PKU6	70 (Group1)	25.53(2.05)	35:35	17.61(2.10) years
	70 (Group2)	23.4(4.79)	35:35	15.99(2.54) years

## Data Availability

The data and materials of this study are available from the corresponding author upon reasonable request.

## Abbreviations

ALE: Activation likelihood estimation
ENM: Exposure network mapping
FDR: False Discovery Rate
GSP: Genome Superstruct Project
LNM: Lesion network mapping
MNI: Montreal Neurological Institute
MRI: Magnetic resonance imaging
sMRI: Structural MRI
